# Effect of signal timing on vehicles’ near misses at intersections

**DOI:** 10.1038/s41598-023-36106-3

**Published:** 2023-06-05

**Authors:** Zubayer Islam, Mohamed Abdel-Aty, Amrita Goswamy, Amr Abdelraouf, Ou Zheng

**Affiliations:** Department of Civil, Environmental and Construction Engineering, University of Central Florida, Orlando, FL 32816 USA

**Keywords:** Engineering, Civil engineering

## Abstract

Driving characteristics often vary between the different states of the signal. During red and yellow phase, drivers tend to speed up and reduce the following distance which in turn increases the possibility of rear end crashes. Intersection safety, therefore, relies on the correct modelling of signal phasing and timing parameters, and how drivers respond to its changes. This paper aims to identify the relationship between surrogate safety measures and signal phasing. Unmanned aerial vehicle (UAV) video data has been used to study a major intersection. Post encroachment time (PET) between vehicles was calculated from the video data as well as speed, heading and relevant signal timing parameters such as all red time, red clearance time, yellow time, etc. Random parameter ordered logit model was used to model the relationship between PET and signal timing parameters. Overall, the results showed that yellow time and red clearance time is positively related to PETs. The model was also able to identify certain signal phases that could be a potential safety hazard and would need to be retimed by considering the PETs. The odds ratios from the models also indicate that increasing the mean yellow and red clearance times by one second can improve the PET levels by 10% and 3%, respectively.

## Introduction

Driver behavior is an important element of road safety which indicates how an individual vehicle behaves due to the driving scene and surrounding environment. The presence of signalized intersections can affect how a person drives. In this study, the authors have used a quantitative surrogate safety measure to model the driving behavior from a safety standpoint: post encroachment time (PET) which can be considered as the temporal gap between two vehicles. Low PET indicates that the lagging vehicle is following too closely which can result in a rear end crash. Therefore, accepting low gaps can be an indicator of risky driving behavior. The authors have investigated whether this behavior can be modelled with respect to signal timing.

Moreover, traffic analysis from a safety point of view has largely relied on crash data. Various statistical methods and machine learning methods have been implemented to understand proactive natures of crash enabling real time prediction of these events. Countermeasures have been developed based on accident data as well. However, crash data can be rare events and there are notable shortcomings of these types of data such as incorrect reasoning, subjectivism, inaccurate data, etc.^1, 2^. Moreover, the specific reasoning to a crash can often be factors other than roadway characteristics and traffic features which cannot be modelled using the prediction algorithms in the literature. On the other hand, conflict events are more common and therefore, can help better to understand design flaws of roadway as well as traffic conditions that impacts conflicts. Several previous studies have definitively proven conflict analysis as an alternative to crash analysis with similar results^2–5^. Several metrics has thus been developed to measure conflict such as Time-to-collision (TTC)^6^, time exposed TTC (TET), time integrated TTC (TIT), time-to-lane crossing (TLC)^7^, Post encroachment time (PET), gap time (GT), encoding time (ET), and time advantage (TAdv)^8^, etc.

The surrogate safety measures are usually dependent on exact localization of road users. For example, to calculate TTC, initial location and velocity would be needed. This requires precise GPS locations. An effective way to study an intersection would be with the help of an Unmanned Aerial Vehicle (UAV) that can be then used to extract accurate trajectories at the centimeter level. These are a better alternative than roadside cameras which have distortion of localization at camera edges. UAVs are also known for easy maneuvering, flexibility, and low cost. UAVs have become an emerging video analysis solution at the transportation level in the recent years. It is often augmented with radar and infrared cameras that can provide a bird’s eye view of an intersection including the approaches. In this study, an intersection was analyzed with respect to PET from the data available through UAV. The signal timing at that instant was also captured. The purpose of this study was to analyze the interaction of safety events and relate it to the signal states.

## Literature review

Traffic safety at intersections has been shown to be dependent on signal timing at that intersection. For example, altering signal phases can better or worsen intersection safety^9^. Several studies have found that there is a direct relation between signal timings and crashes. After any retiming of signals, a crash reduction factor is also estimated but few studies have also reported that there were no significant relationships^10^. Guo, Wang^11^ showed that adaptive intersections experienced fewer crashes than isolate ones. The study was extensive and included over 170 intersections in Florida, USA but the results were based on signal timing sheets only since real traffic data was not available. Midenet, Saunier^12^ evaluated signal safety by measuring the exposure to lateral collisions using video feed. Approach level data from traffic detectors including speed, volume was found to be associated with significant crash risk^13^. It was also reported in this study that longer green time for left turn, higher green ratio can improve the safety at intersections. The main limitation of all the studies is that crash events are usually rare and therefore, these studies would only rely on the spatial relationship between crash events and traffic parameters. It has been shown in several studies that the temporal relationship need to be included as well since traffic parameters and signal timing would vary largely throughout the day and even across days^14–16^. Moreover, there are notable shortcomings of these types of police reported crash data such as incorrect reasoning, subjectivism, inaccurate data, etc.^1, 2^. Additionally, there is the moral dilemma of waiting for fatalities to happen before taking an appropriate countermeasure making it a reactive approach. Crash events are also rare, and it takes a long time to study a location or conduct a before-after study. Surrogate safety measures provide an alternate and proactive methodology that does not require much time and solves the moral dilemma to a great extent. Several studies have also shown that it can significantly correlate to crashes and can mostly be used as an alternative^2–5, 17^.

Using surrogate safety measures for signal timing was first proposed by Stevanovic, Stevanovic^18^. The study proposed the integration of optimization and surrogate safety measure assessment at the microscopic level considering both the safety and efficiency. Network wide optimization was also studied in recent time^19^. This work also incorporates simulation and surrogate safety measures to find optimal solution using a model calibrated from real-world data. The influence of signal phasing on the safety and traffic smoothness was also studied^20, 21^. It was also shown that optimization of the left turn waiting zones would improve capacity without degrading traffic flow^22^ while Lin and Huang^23^ improved both at signal coordination level across multiple intersections. All the studies have relied on simulation software such as VISSIM to model traffic signals and safety. While some studies calibrate the models based on real traffic flow, the ground data can be significantly different than the simulation. This work addresses this research gap and uses real-world data from UAVs to evaluate signal timing based on Post Encroachment Time (PET). The main objective of this work was to evaluate the impact of all-red time, red clearance time, red time, yellow time and green time on the surrogate safety measures based on real-world data. These can also help relevant authorities to understand intersection traffic with respect to PET and gain insight whether the signal timing need optimization or not. Moreover, the odds ratio was also calculated to show that one second increase of yellow and red clearance time will help to increase the PET level thereby improving the safety condition of the intersection.

## DATA preparation

### Trajectory data

The vehicle trajectories provided by the CitySim dataset^24^ were utilized to identify, process, and analyze PET conflicts in this study. The CitySim dataset is composed of top-view drone-video-based vehicle trajectories. The authors identified vehicle trajectories using mask-RCNN and subsequently extracted and exported rotation-aware bounding boxes. The process involves an extensive five-step pipeline: video stabilization, object filtering, video stitching, detection and tracking, and enhanced error filtering. Video stabilization was obtained through Scale-Invariant Feature Transform (SIFT) algorithm. Gaussian-mixture-based algorithm was used to filter background objects. Afterwards, object detection algorithm Mask R-CNN was used to obtain rotating bounding boxes. Finally, any remaining errors were filtered using human-in-the-loop. Each frame was checked manually to ensure the exactness of the bounding boxes.

**Figure 1:**
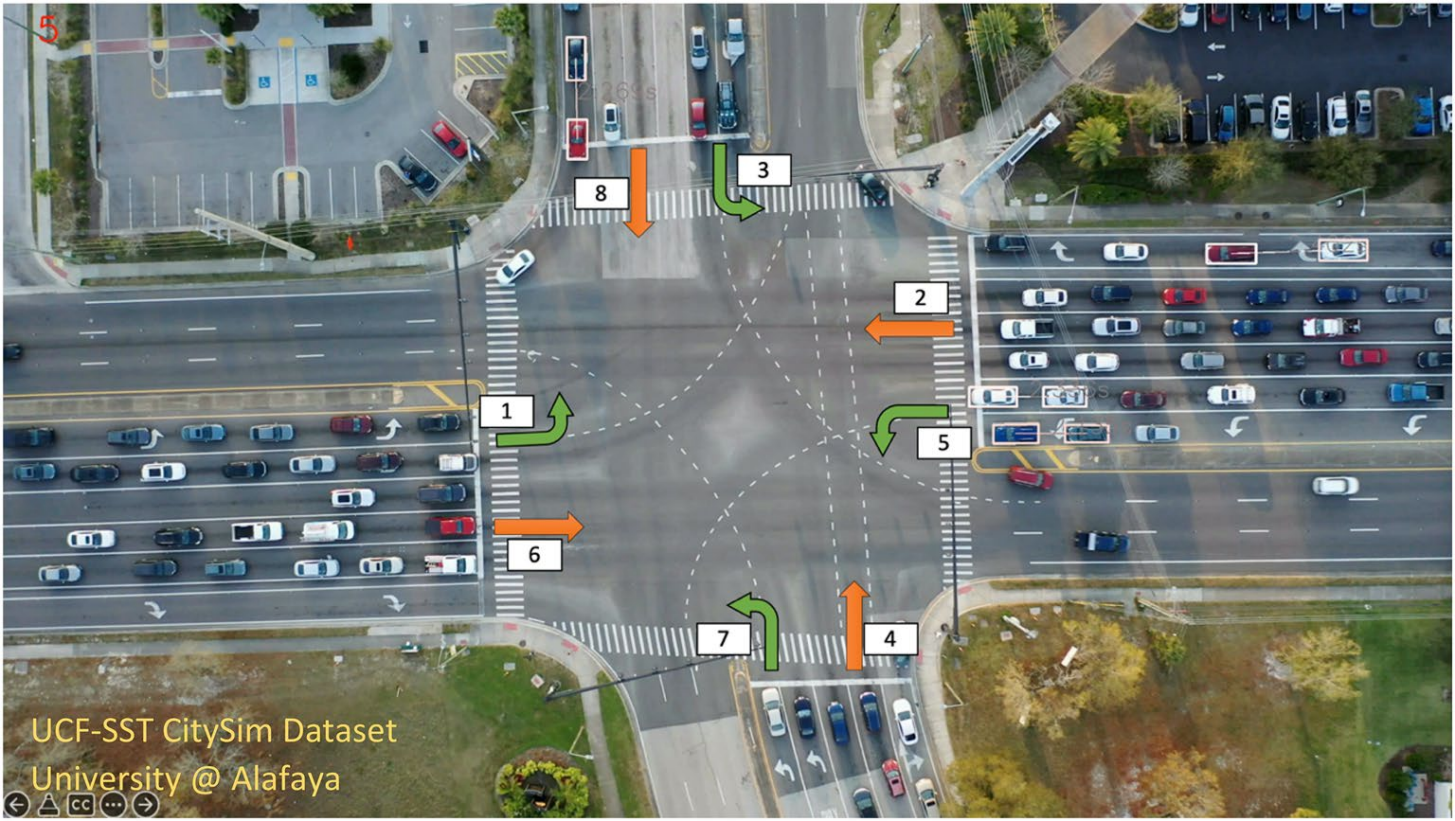
Study intersection location showing the different phases.

The dataset contains vehicle trajectories sampled at 30 frames per second. For each trajectory point, the dataset provides four bounding box positions, speed, and heading. In this work, the University@Alafaya intersection location was selected for development, evaluation, and analysis. The intersection geometry is illustrated in Fig. 1. It is a signalized intersection between Alafaya Trail (9 lanes) and University Boulevard (9 lanes). The utilized trajectories were extracted from a video recorded on a weekday between 5:40 PM and 6:40 PM (afternoon peak). A total of 4871 vehicles passed through the intersection during that period of time. The different phases for each traffic direction are also shown in Fig. 1. There are three through lanes for each of the phases 2,4,6 and 8 while two left turning lanes for phases 1,3,5 and 7. The approach 4 does not have any exclusive right turn lanes while the other through phases all have an exclusive right turn lane.

### Post encroachment time (PET)

**Figure 2:**
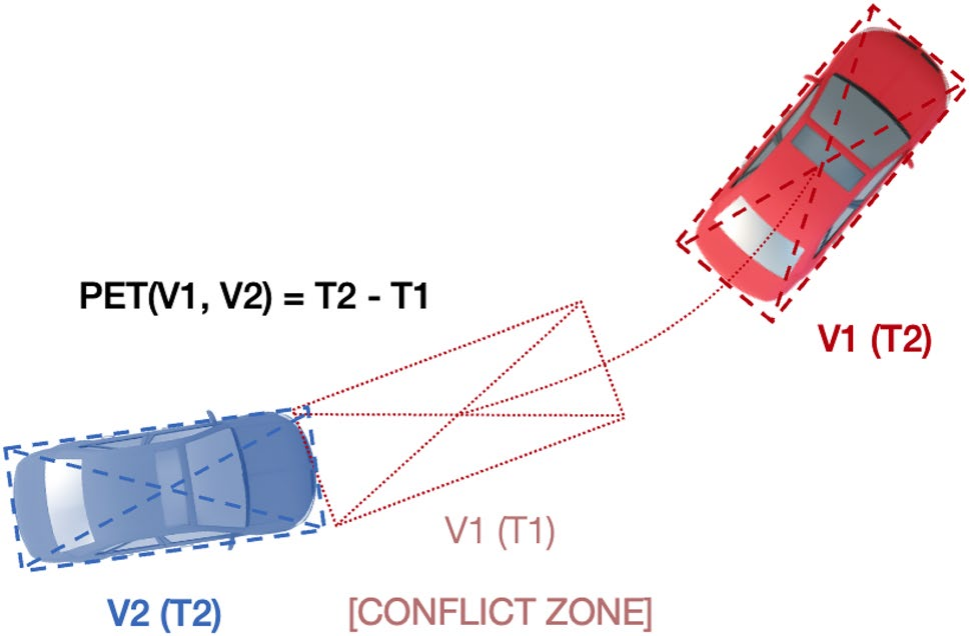
Example PET calculation between leading vehicle (V1) and lagging vehicle (V2) in the time period between (T1) – (T2) in the conflict zone V1(T1).

Post Encroachment Time is a conflict indicator that serves as a surrogate safety measure. Figure 2 depicts an example PET conflict between two vehicles at a single timestep. PET measures the period of time between a leading vehicle leaving a particular location and a lagging vehicle arriving at the same location. In this scenario, the location where both vehicles interact is dubbed the conflict zone. The PET conflict indictor generates a sequence of PET values that describe the serial interaction between two vehicle trajectories under observation. A PET value exists in the generated PET sequence as long as the lagging vehicle remains in a conflict zone. Otherwise, the PET value at a timestep where no encroachment occurs is undefined.

In this research effort, the PET values were computed using the rotation-aware vehicle bounding boxes provided by the CitySim Dataset. At each timestep, and for each possible pair of vehicles, the PET value was measured between the moment a lagging vehicle bounding box intersects with a leading vehicle’s previous bounding box location (i.e., the lagging vehicle intersects with the conflict zone as described in Fig. 2). For each pair of vehicles, an output PET sequence that describes their interaction was generated. The selected timestep was 1/3 s (3 Hz). PET values under 5 s were recorded.

Table 1 describes the PET conflicts extracted from the study area. When sampled at 3 Hz, a total of 193,000 PET conflicts under 5 s were captured in the study area. Additionally, Table 1 reports the minimum PETs (minPETs). The minPET is defined as the minimum PET recorded between 2 vehicle trajectories. It describes the single most hazardous moment between unique vehicle pairs. Table 1 indicates that, during the recorded time, 717 unique vehicle pairs recorded a minPET under 1 s, and 7345 unique vehicle pairs encountered a minPET conflict under 5 s.

**Table 1:** Number of PET conflicts and minPET values for different thresholds.

PET threshold	< 1.0s	< 2.0s	< 3.0s	< 4.0s	< 5.0s
Number of PET conflicts (sampled at 3 Hz)	9 K	62 K	106 K	150 K	193 K
Number of minPETs for unique vehicle pairs	717	2785	4365	5897	7345

**Figure 3:**
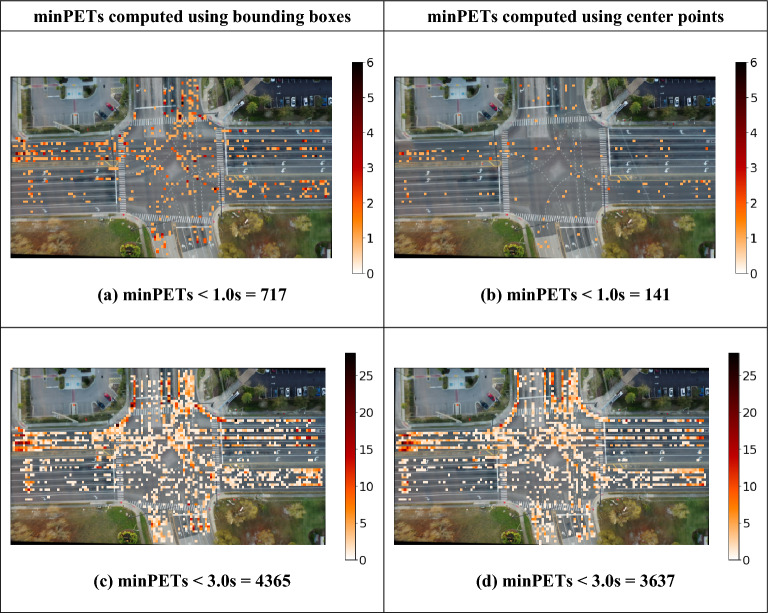
minPET heatmaps computed using bounding boxes versus center points.

Utilizing the vehicle bounding boxes for PET calculation is not common within previous research efforts. Instead, most previous work relied on the trajectories of the center-point-based conflict identification. As illustrated in Fig. 2, the vehicle geometry is essential for robust PET measurement. Center points misrepresent vehicle geometries and lead the conflict identification algorithm to neglect conflicts or underestimate their severity^25^. Figure 3 compares heatmap plots of minPETs recorded in the study intersection using bounding boxes versus center points. It can be clearly observed that the bounding box approach was able to recall more conflicts than the center point method. For a minPET < 1.0 s, the center point method identified 141 compared to 717 conflicts captured by the bounding box. Similarly, for a minPET maximum threshold of 3.0 s, the center point and bounding box methods identified 3637 and 4365 conflicts, respectively. Figure 3 clearly demonstrates the superiority and robustness of the bounding box approach. Furthermore, it indicates that the center point misdetection rate is proportional to the conflict severity, meaning that center-point-based computations fail to capture the most hazardous traffic conflicts.

Five different levels of PET were chosen based on past literature. In a study conducted by Zheng, Ismail^26^, it was found that a PET threshold of 1.5 s exhibited the strongest correlation between crashes and conflicts. Results also indicated that PET thresholds of 1.5 s, 2 s, 2.5 s, and 3 s were all significantly correlated with crashes. Peesapati, Hunter^27^ found through their study using CDF and absolute number of PETs that values less than 1 s and 1.5 s were the most related to crashes, and PETs less than 3 s showed a degrading Pearson Coefficient. Another study by Zheng and Sayed^28^ chose a threshold value of 4 s to analyze extreme values of conflicts only. Based on previous research, PET values less than 1 s or 2 s are considered critical, while those between 2 and 4 s are intermediate, and those between 4 and 5 s are mild conflicts. PETs were preferred over other conflict measures, such as TTC, because TTC assumes a straight-line collision course and is not suitable for intersections with left and right turn motions. PETs, on the other hand, can capture angle/crossing conflicts accurately^29^.

All the different datasets involving PET, speed, heading, and signal timing were merged together to obtain the final dataset. The descriptive statistics of the different variables as well as brief explanation of each variable in the final dataset are shown in Table 2. The various signal timing such as red, green, yellow, etc. are modelled as a countdown timer to understand the impact of the time remaining of a phase on PETs.

**Table 2:** Variable statistics.

Feature	Description	Count	Mean	SD	Min	Max
PET level	1, if 0.3 s < PET < = 1 s	78,859	3.3	1.18	1	5
	2, 1 s < PET < = 2 s					
	3, 2 s < PET < = 3 s					
	4, 3 s < PET < = 4 s					
	5, 4 s < PET < = 5 s					
Distance (ft)	Spatial gap between two vehicles	78,859	13.98	1.53	0	15
red_clearance (s)	Red clearance time remaining at the end of each phase	78,859	0.2	0.76	0	4.9
all_red (s)	All red time remaining at the end of each cycle	78,859	0.02	0.22	0	3.9
Red (s)	Red time remaining	78,859	6.11	25.28	− 1	175.9
Yellow (s)	Yellow time remaining	78,859	0.01	0.61	− 1	4.9
Green (s)	Green time remaining	78,859	19.25	16.8	− 1	85.3
Phase 1	1, if phase is active, 0, otherwise	78,859	0.13	0.34	0	1
Phase 2		78,859	0.32	0.46	0	1
Phase 3		78,859	0.12	0.33	0	1
Phase 4		78,859	0.02	0.15	0	1
Phase 5		78,859	0.03	0.19	0	1
Phase 6		78,859	0.09	0.29	0	1
Phase 7		78,859	0.11	0.32	0	1
Phase 8		78,859	0.06	0.24	0	1
Speed (mph)	Current vehicle Speed	78,859	19.48	10.43	0	59.98
Heading (degrees)	Direction of travel	78,859	190.81	100.83	0	360
Lane	Lane information for any PET	78,859	28.3	10.31	3	35
Volume	Number of vehicles per 5 min	78,859	49.1	11.68	19	86
Intersection	1, if the vehicle is at intersection 0, otherwise	78,859	0.6	0.49	0	1
speeding_prop	$\frac{speed-speed limit}{speed limit}$ for leading vehicle	78,859	− 0.45	0.24	− 0.89	0.33
Movement	Location of the vehicle 0, left turning lane 1, through lane 2, at intersection	78,859	1.48	0.7	0	2

**Figure 4:**
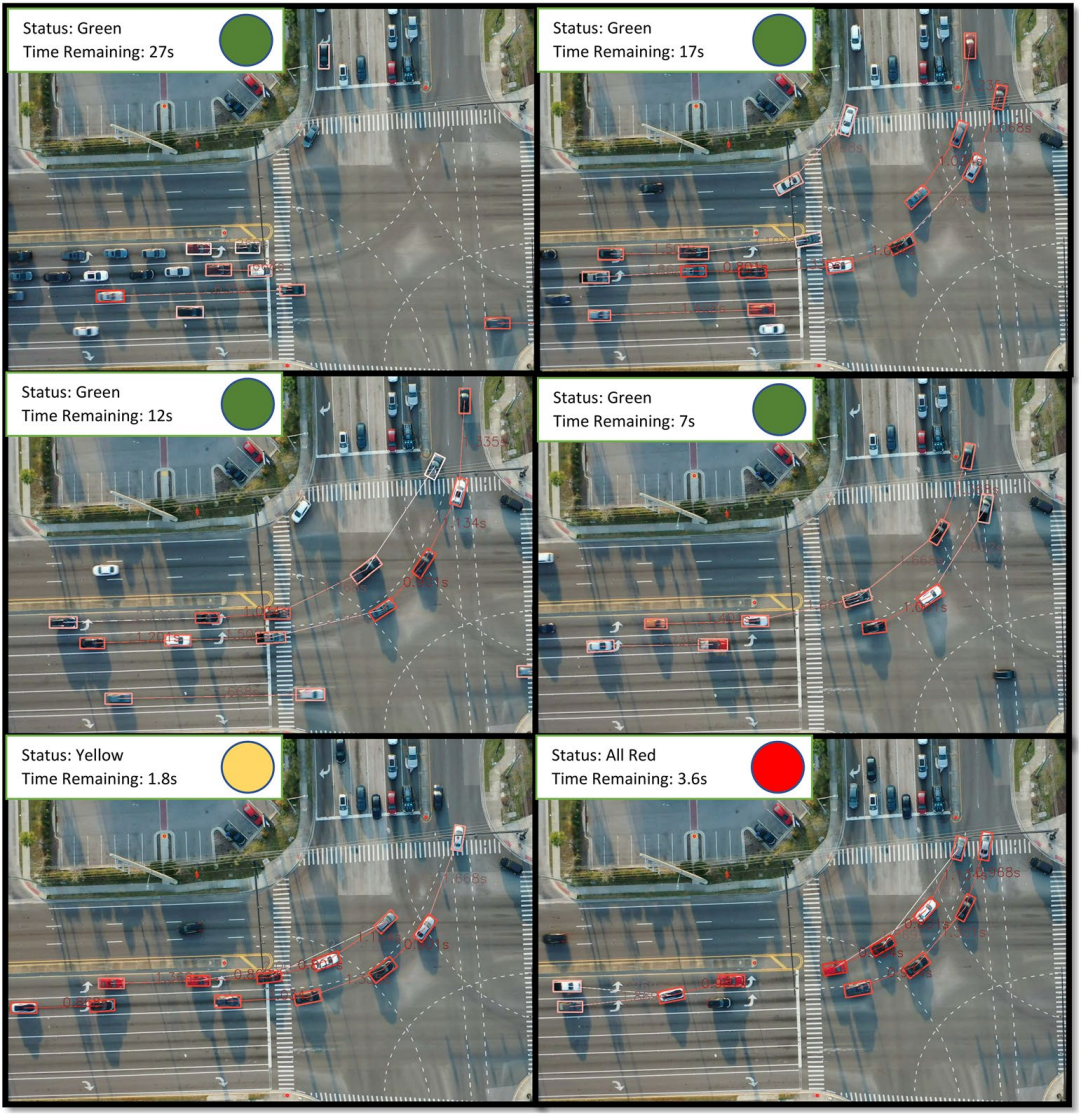
Sequence of signal timing events showing decrease in PET for phase 1.

A sample case of changing PETs towards the end of a cycle is shown in Fig. 4. The PETs between interacting vehicles are shown in the figure. The lower the PET, the redder is the bounding box indicating high severity. It can be noted that as the phase turns green the vehicles start to move with PETs between 1.5 to 2 s. As the phase turns from yellow to red, the PET even lowers to 0.8 s as drivers try to clear the intersection.

## Model

### Random parameter ordered logit model

Random parameters logit model is a logit model for which the parameters are assumed to vary from one case to another. It is therefore a model that takes the heterogeneity of the population into account. In this study five levels of PET were considered.

We follow Milton et al. (2008) and Washington et al. (2011), and start with

$${U}_{ij}={\beta }_{j}{X}_{ij}+{\varepsilon }_{ij}$$

where ${U}_{ij}$ is a function determining the PET level i on individual PET for observaions, ${X}_{ij}$ is a vector of explanatory variables; βi is a vector of estimable parameters for outcome i which may vary across observations, and ${\varepsilon }_{ij}$ is the error term which is assumed to be generalized extreme value distributed (McFadden, 1981).

In order to develop random parameter models, we consider the following latent process as described by Sarrias Mauricio, 2016

1 $$\begin{aligned} y_{it}^{*} & = x_{it}^{T} \beta_{i} + \epsilon_{it,} i = 1, \ldots ,n;\;t = 1, \ldots T_{i} \\ \beta_{i} & \sim g\left( {\beta_{i} \left| \theta \right|} \right), \\ \end{aligned}$$

where ${y}_{it}^{*}$ is a latent (unobserved) process for individual i in period t, ${x}_{it}$ is a vector of covariates, and ${\epsilon }_{it}$ is the error term.

Note that the conditional probability density function (PDF) of the latent process $f\left({y}_{it }^{*}\left|{x}_{it,} {\beta }_{i}\right|\right)$ is determined once the nature of the observed ${y}_{it}$ and the population PDF of ${\epsilon }_{it}$ is known. If ${y}_{it}$ is binary and ${\epsilon }_{it}$ is distributed as normal, then the latent process becomes the traditional probit model; if ${y}_{it}$ is an ordered categorical variable and $\varepsilon_{it}$ is logistically distributed, then the traditional ordered logit model arises. Formally, the PDF for binary, ordered, and Poisson model are, respectively

2 $$\begin{aligned} f\left( {y_{{\left\{ {it} \right\}}}^{*} \left| {x_{{\left\{ {it} \right\}}} ,\beta_{i} } \right|} \right) & = \left[ {F\left( {x_{{\left\{ {it} \right\}}}^{T} \beta_{i} } \right)} \right]^{{y_{{\{_{it} }} }} \left[ {1 - F\left( {x_{{\left\{ {it} \right\}}}^{T} \beta_{i} } \right)} \right]^{{1 - y_{it} }} \\ & \quad = \mathop \prod \limits_{j = 1}^{J} \left[ {F\left( {k_{j} - x_{it}^{T} \beta_{i} } \right) - F\left( {k_{j - 1} - x_{it}^{T} \beta_{i} } \right)} \right]^{{y_{itj} }} \\ & \quad = \frac{1}{{y_{it} !}}\exp \left[ { - \exp \left( {x_{it}^{T} \beta_{i} } \right)} \right]\exp \left( {x_{it}^{T} \beta_{i} } \right)^{{y_{it} }} \\ \end{aligned}$$

For the binary and ordered models, F (·) represents the cumulative distribution function (CDF) of the error term, which $F\left(\epsilon \right)=\Phi \left(\epsilon \right)$ for probit and $F\left(\epsilon \right)=\Gamma \left(\epsilon \right)$ for logit. For the ordered model, ${\kappa }_{j}$ represents the threshold for alternative j = 1, . . ., J − 1, such that ${\kappa }_{0 }=-\infty$ and ${\kappa }_{0 }=\infty$.

In the structural model given by Eq. (1), we allow the vector coefficient ${\beta }_{i}$ to be different for each individual in the population. In other words, the marginal effect on the latent dependent variable is individual-specific. Nevertheless, we do not know how these parameters vary across observations. All we know is that they vary according to the population PDF $g\left({\beta }_{i }\left| \theta \right|\right)$ where $\theta$ represents the moments of the distribution such as the mean and the variance, which must be estimated. A fully parametric model arises once $g\left({\beta }_{i }\left| \theta \right|\right)$ and the distribution of $\epsilon$ are specified.

For simplicity in notation, assume that the coefficient vector is independent normal distributed, so that ${\beta }_{k }\sim N\left({\beta }_{k }, {\sigma }_{k}^{2}\right)$ for the k-th element in i. Note that each coefficient can be written as ${\beta }_{ki }= {\beta }_{k }+ {\sigma }_{k }{\omega }_{i}$ where ${w}_{i }\sim$ N(0, 1), or in vector form as ${\beta }_{i }= \beta + L {\omega }_{i}$, where L is a diagonal matrix that contains the standard deviation parameters, ${\sigma }_{k}$. All the information about the individual heterogeneity for each individual attribute is captured by the standard deviation parameter ${\sigma }_{k}$. If ${\sigma }_{k}$ = 0, then the model is reduced to the fixed parameter model, but if it is indeed significant then it would reveal that the relationship between ${x}_{itk}$ and ${y}_{it}$ is heterogeneous and focusing just on the central tendency k alone would veil useful information. It is useful to note that the random effect model is a special case in which only the constant is random.

Some measures of goodness-of-fit including Log-Likelihood and Akaike Information Criterion (AIC) were used to find the best fitted model. The best fitted model that displayed the maximum value of the log-likelihood function was chosen to obtain the parameter estimates that made the data most likely. AIC value was used to compare the performances of the GLMs. The preferred model is the one with the minimum AIC value. The AIC value can be evaluated using:

$${\text{AIC}} = {2} {\text{k}} - {\text{2ln }}({\text{L}})$$

where k = The number of estimated parameters in the model, L = The maximum value of the likelihood function for the model.

The Bayesian Information Criterion (BIC) values were also calculated to conclude the best model that describes the relationship between each crash type and the explanatory variables. The AIC introduces a penalty term that is represented by the parameter number in the AIC. The BIC introduces the penalty term as a combination between the parameter number and sample size^30^.

### Random parameter ordered logit model with observed heterogeneity

This extension of the ordered logit model, allows the coefficients to be corelated. The covariance matrix of the random parameters can be shown as $L{L}^{T} =\sum$, where L is a lower triangular matrix. If $\prod$ is a matrix of parameters, ${s}_{i}$ is a vector of covariates not varying in time and $\omega \sim N(\mathrm{0,1})$, the parameter vector, its mean and covariance can be written as

$${\beta }_{i}=\beta +\prod {s}_{i} + L{\omega }_{i}$$

$$E({\beta }_{i}) = \beta +\prod {s}_{i} + LE(\omega ) = \beta +\prod {s}_{i}$$

$$VAR({\beta }_{i}) =E({L\omega (\omega L)}^{T})=LE(\omega {\omega }^{T}){L}^{T}=L{L}^{T}=\sum$$

## Results

The results from the Random Parameter Logit Model and that with heterogeneity are shown in Table 3 and the conclusions are presented in the subsections. It is important to note here that the signal times are modelled as a countdown timer. For example, a yellow time of 4 s means that the signal state is currently yellow and has 4 s remaining. The reason the authors decided to model the signal timings as a countdown timer is that most of the times the drivers would try to speed up or slow down to comply with the end of the signal timings. Since AIC is used for selecting prediction model and BIC is used for model explanation^31^, the authors have chosen the appropriate model based on BIC values. The model with heterogeneity in means had better BIC values except for yellow time. Thus, the random parameter ordered logit model is suggested for yellow time and that with heterogeneity in means is suggested for all other signal times. The signs of the different variables were identical in both the models.

**Table 3:** Models for PET levels on signal timing.

Yellow time											
Random parameters ordered logit models						Random parameters ordered logit model with heterogeneity in means					
Coefficients:	Estimate	Odds ratio	Std. error	z-value	Pr( >|z|)	Coefficients:	Estimate	Odds ratio	SE	z-value	Pr( >|z|)
No. of Obs = 2955, Log Likelihood = − 3667, AIC = 7361, BIC = 7445						No. of Obs = 2955, Log Likelihood = − 3658, AIC = 7351, BIC = 7459					
Constant	4.685	–	0.884	5.301	0***	Constant	4.603		0.824	5.584	0.000***
Distance	− 0.175	0.839	0.048	− 3.661	0***	Distance	− 0.198	0.820	0.050	− 3.979	0.000***
Yellow	0.151	1.163	0.035	4.359	0***	Mean: yellow	0.104	1.109	0.043	2.434	0.015*
						SD: yellow	0.363	1.437	0.031	11.528	0.000***
Phase 3	0.728	2.071	0.321	2.269	0.023*	Phase 3	1.257	3.513	0.299	4.196	0.000***
Phase 5	− 0.897	0.408	0.474	− 1.893	0.058	Phase 5	− 0.461	0.631	0.337	− 1.367	0.172
Phase 6	2.004	7.416	0.55	3.641	0***	Phase 6	3.262	26.093	0.808	4.035	0.000***
Phase 7	1.444	4.238	0.288	5.022	0***	Phase 7	1.650	5.205	0.362	4.552	0.000***
Phase 8	2.032	7.628	1.005	2.022	0.043*	Phase 8	2.190	8.940	0.733	2.989	0.003**
Mean. volume	0.01	1.01	0.013	0.745	0.456	Mean. volume	0.022	1.023	0.013	1.782	0.075
SD volume	0.056	1.058	0.003	18.97	0***	SD volume	0.051	1.052	0.002	24.037	0.000***
						Heterogeneity in mean: volume * yellow	− 0.001	0.999	0.002	− 0.707	0.480
Kappa.1	3.853	–	0.117	32.892	0***	Kappa.1	3.862	–	0.115	33.700	0.000***
Kappa.2	5.298	–	0.13	40.651	0***	Kappa.2	5.339	–	0.130	41.144	0.000***
Kappa.3	7.004	–	0.15	46.577	0***	Kappa.3	7.080	–	0.151	46.880	0.000***

**All red time**											
No. of Obs = 805, Log Likelihood = − 683.8, AIC = 1399.564, BIC = 1474.618						No. of Obs = 805, Log Likelihood = − 651.1, AIC = 1342, BIC = 1435					
Constant	14.475	–	1.162	12.451	0***	Constant	13.571		1.199	11.319	0.000***
Speeding prop	3.956	52.274	0.732	5.404	0***	Speeding_prop	5.040	154.526	0.563	8.949	0.000***
All red	− 0.558	0.572	0.124	− 4.515	0***	Mean. all_red	0.172	1.188	0.129	1.333	0.183
						SD. all_red	1.252	3.496	0.111	11.285	0.000***
						Distance	0.111	1.117	0.054	2.044	0.041*
Phase 1	− 2.573	0.076	0.57	− 4.514	0***	Phase 1	− 1.843	0.158	0.568	− 3.244	0.001**
Phase 2	3.644	38.238	0.605	6.027	0***	Phase 2	1.792	6.001	0.410	4.374	0.000***
Phase 3	− 1.262	0.283	0.573	− 2.202	0.028*	Phase 3	− 2.431	0.088	0.482	− 5.040	0.000***
Phase 4	4.986	146.288	1.332	3.743	0***	Phase 4	1.133	3.105	0.944	1.200	0.230
Phase 5	4.196	66.43	1.122	3.739	0***	Phase 5	1.593	4.917	0.598	2.665	0.008**
Phase 7	4.058	57.855	0.861	4.711	0***	Phase 8	− 2.607	0.074	1.067	− 2.444	0.015*
Mean. volume	− 0.089	0.915	0.018	− 5.022	0***	Volume	− 0.074	0.929	0.013	− 5.742	0.000***
SD volume	0.117	1.124	0.007	15.714	0***	mean. intersection	7.445	1711.371	1.370	5.435	0.000***
						SD intersection	4.824	124.454	0.322	14.999	0.000***
						Heterogeneity in mean: intersection.all_red	− 2.648	0.071	0.396	− 6.684	0.000***
Kappa.1	4.96	–	0.438	11.316	0***	Kappa.1	5.22277	–	0.44709	11.682	0.000***
Kappa.2	8.259	–	0.515	16.039	0***	Kappa.2	8.71902	–	0.54801	15.91	0.000***
Kappa.3	11.026	–	0.596	18.487	0***	Kappa.3	11.38398	–	0.61409	18.538	0.000***

Red clearance											
No. of Obs = 6785, Log Likelihood = − 8881, AIC = 19,792, BIC = 19,915						No. of Obs = 6785, Log Likelihood = − 8956, AIC = 17,952, BIC = 18,088					
Constant	8.072	–	0.574	14.058	0***	Constant	7.327		0.418	17.511	0.000***
Speeding prop	0.421	1.523	0.159	2.654	0.008**	Speeding_prop	0.156	1.169	0.167	0.933	0.351
Distance	− 0.155	0.856	0.023	− 6.672	0***	Distance	− 0.158	0.854	0.024	− 6.544	0.000***
Red clearance	0.033	1.033	0.023	1.443	0.149	Mean. red_clearance	0.034	1.034	0.028	1.225	0.220
						SD. red_clearance	0.513	1.671	0.021	23.887	0.000***
Intersection	− 0.338	0.714	0.084	− 4.003	0***	Mean. intersection	1.825	6.200	0.297	6.138	0.000***
						SD. intersection	1.646	5.186	0.091	18.054	0.000***
Phase 1	− 0.701	0.496	0.413	− 1.696	0.09	Phase 1	− 0.618	0.539	0.395	− 1.565	0.118
Phase 2	− 1.03	0.357	0.209	− 4.937	0***	Phase 2	− 0.701	0.496	0.138	− 5.088	0.000***
Phase 3	− 1.821	0.162	0.605	− 3.01	0.003**	Phase 3	− 1.437	0.238	1.380	− 1.041	0.298
Phase 4	− 5.175	0.006	0.703	− 7.36	0***	Phase 4	− 4.856	0.008	0.629	− 7.726	0.000***
Phase 5	− 3.126	0.044	0.329	− 9.492	0***	Phase 5	− 2.391	0.092	0.203	− 11.804	0.000***
Phase 6	− 1.471	0.23	0.23	− 6.402	0***	Phase 6	− 0.820	0.441	0.192	− 4.272	0.000***
Phase 7	− 1.488	0.226	0.218	− 6.82	0***	Phase 7	− 1.528	0.217	0.174	− 8.775	0.000***
Phase 8	− 1.106	0.331	0.319	− 3.466	0.001***	Phase 8	− 1.728	0.178	0.389	− 4.447	0.000***
Mean. volume	− 0.001	0.999	0.01	− 0.063	0.95	Volume	0.005	1.005	0.004	1.388	0.165
SD volume	0.042	1.043	0.002	25.986	0***						
						Heterogeneity in mean: intersection.red_clearance	− 0.532		0.071	− 7.533	0.000***
Kappa.1	3.514	–	0.083	42.558	0***	Kappa.1	3.456	–	0.080	43.082	0.000***
Kappa.2	4.85	–	0.089	54.498	0***	Kappa.2	4.754	–	0.086	55.066	0.000***
Kappa.3	6.514	–	0.098	66.606	0***	Kappa.3	6.380	–	0.095	67.419	0.000***

Red time											
No. of Obs = 7112, Log Likelihood = − 10,150, AIC = 20,334, BIC = 20,451						No. of Obs = 7112, Log Likelihood = − 10,130, AIC = 20,301, BIC = 20,432					
Constant	4.198	–	0.222	18.95	0***	Constant	4.982		0.284	17.577	0.000***
Speeding prop	1.398	4.049	0.105	13.264	0***	Speeding_prop	1.471	4.354	0.117	12.545	0.000***
Distance	0.096	1.101	0.011	9.036	0***	Distance	0.104	1.110	0.011	9.114	0.000***
Red	− 0.004	0.996	0.001	− 7.579	0***	Mean. red	− 0.014	0.986	0.003	− 5.666	0.000***
						SD. red	0.007	1.007	0.001	7.238	0.000***
Intersection	− 1.043	0.352	0.07	− 14.912	0***	Intersection	− 1.101	0.333	0.078	− 14.075	0.000***
Phase 2	0.371	1.45	0.089	4.158	0***	Phase 2	0.429	1.535	0.095	4.531	0.000***
Phase 4	0.639	1.894	0.163	3.921	0***	Phase 4	0.617	1.853	0.176	3.504	0.000***
						Phase 5	− 0.245	0.783	0.134	− 1.826	0.068*
Phase 6	0.652	1.92	0.134	4.868	0***	Phase 6	0.769	2.158	0.142	5.432	0.000***
Mean. volume	− 0.011	0.989	0.002	− 4.674	0***	Mean. volume	− 0.023	0.977	0.004	− 6.216	0.000***
SD volume	0.013	1.013	0.003	4.616	0***	SD volume	0.014	1.015	0.003	4.622	0.000***
						Heterogeneity in mean: volume.red	0.0002	1.000	0.000	4.212	0.000***
Kappa.1	2.4	–	0.084	28.496	0***	Kappa.1	2.568	–	0.108	23.883	0.000***
Kappa.2	3.659	–	0.122	30.101	0***	Kappa.2	3.920	–	0.159	24.668	0.000***
Kappa.3	4.859	–	0.157	30.943	0***	Kappa.3	5.197	–	0.206	25.172	0.000***

Green time											
No. of Obs = 62,416, Log Likelihood = − 91,730, AIC = 183,497, BIC = 183,651						No. of Obs = 62,416, Log Likelihood = − 91,730, AIC = 183,449, BIC = 183,631					
Constant	4.767	–	0.092	51.701	0***	Constant	4.870		0.111	43.990	0.000***
Speeding prop	0.85	2.34	0.033	25.571	0***	Speeding_prop	0.856	2.353	0.033	25.624	0.000***
Distance	− 0.082	0.921	0.005	− 14.983	0***	Distance	− 0.082	0.921	0.005	− 15.020	0.000***
Green	0.019	1.02	0.001	28.633	0***	Mean. green	0.015	1.015	0.002	6.178	0.000***
						SD. green	0.000	1.000	0.001	0.205	0.837
Intersection	− 0.272	0.762	0.016	− 16.767	0***	Intersection	− 0.273	0.761	0.016	− 16.785	0.000***
Phase 2	− 0.265	0.767	0.027	− 9.951	0***	Phase 2	− 0.254	0.776	0.027	− 9.301	0.000***
Phase 4	− 0.922	0.398	0.05	− 18.607	0***	Phase 4	− 0.897	0.408	0.051	− 17.507	0.000***
Phase 5	0.146	1.157	0.046	3.202	0.001**	Phase 5	0.166	1.181	0.047	3.525	0.000***
Phase 6	− 0.641	0.527	0.035	− 18.238	0***	Phase 6	− 0.642	0.526	0.035	− 18.245	0.000***
Phase 7	0.174	1.19	0.032	5.374	0***	Phase 7	0.176	1.193	0.032	5.440	0.000***
Phase 8	− 0.606	0.546	0.039	− 15.69	0***	Phase 8	− 0.594	0.552	0.039	− 15.112	0.000***
Mean. volume	− 0.001	0.999	0.001	− 1.206	0.228	Mean. volume	− 0.003	0.997	0.001	− 2.037	0.042*
SD volume	0.006	1.006	0.001	4.394	0***	SD volume	0.006	1.006	0.001	4.484	0.000***
						Heterogeneity in mean: volume. green	0.0001	1.000	0.000	1.665	0.096
Kappa.1	2.511	–	0.024	103.157	0***	Kappa.1	2.512	–	0.024	102.981	0.000***
Kappa.2	3.531	–	0.032	110.856	0***	Kappa.2	3.533	–	0.032	110.595	0.000***
Kappa.3	4.712	–	0.041	115.072	0***	Kappa.3	4.715	–	0.041	114.763	0.000***

It was seen that the random parameters models performed better than the fixed effects models as the AIC and BIC values of the random parameter models were much lower than those of the fixed effects models. The study evaluated the effect of signal times on PET levels. Thus, five models for different signal times (yellow, all red, red clearance, red and green) were performed to ascertain its effects on PETs. As mentioned before, PETs less than 1 was indicated as level 1, and the data had five levels of PET, with PET values ranging from 0.3 s to 4.97 s. Other independent variables in the models were the different phases for the signal cycle, phase 1 through 8, where phases 2,4,6,8 were for through and right turn and phases 1,3,5,7 were left turning ones. In summary, the negative signs of the coefficients in Table 3 reduce PET levels (increases conflict severity) while positive signs increase PET levels (decreases conflict severity).

### PET for intersection

PET values also have different effects when vehicles are inside the intersection verses when they were at the approach. It can be seen that for the models of red clearance, red and green timings, the intersection indicator variable was significant and the coefficient being negative indicates that in intersections the PET levels are in general low meaning the vehicles have tendency to maintain small gaps between them, which can in turn be a risky situation.

### Yellow time vs PET

The overall yellow time is positively related to the different PETs. The lower the yellow time, the lower the PET which shows that the vehicles tend to follow each other closely towards the end of the yellow phase. The variable phase 5 shows that when the yellow for this phase is active, there are low PETs. This essentially indicates a probable issue with the length of the yellow time. The other phases that came out to be significant have the opposite relationship and can be interpreted to be safer.

### All red time vs PET

All red time is negatively correlated to PET. This shows that the vehicles that enter the intersection at the end of yellow have lower PET since they are essentially trying to clear the intersection. Together with the yellow time and all red time, it can be concluded that there are lower PETs at the boundary of yellow and all red time. The variables phase 1 and phase 3 have negative sign meaning that when the all red of these phases are active, there are lower PETs resulting in an unsafe state. This also helps to conclude the visualization in Fig. 2, where we see a snapshot of the traffic state for phase 1 at all red time of 1.8 s.

### Red clearance time vs PET

The red clearance time was not significant in the model but from the individual red clearance time per phase it is noted that the relationship is negative meaning that each of the clearance times experience lower PET. This can also be noted as a potential safety condition that will require careful signal timing optimization. Almost all phases except phase 1, were found to be significant.

### Red time, green time vs PET

It can be seen that increase green signal times of a cycle have positive signs indicating potential for increasing PET level. Which in turn signifies that increase in these timings have potential to increase PET values between vehicles and increasing safety by reducing probability of conflict leading to rear end crashes. On the other hand, increase in red times, influences the PET levels to decrease meaning that the increase in these timings have potential to decrease the PET. This is expected since as red time is increased, the vehicles are stopped at the approach and therefore have no conflicts.

### Speeding proportion vs PET

Increase of speed of the vehicle also leads to an increase in PET. The speeding proportion is calculated for the leading vehicle and as such once this vehicle speeds, the distance between interacting vehicles increases thus increasing PET.

### Odds ratio

Standard interpretation of the ordered logit coefficient is that for a one unit increase in the predictor, the response variable level is expected to change by its respective regression coefficient in the ordered log-odds scale while the other variables in the model are held constant. Thus, we calculate Odds ratio. Odds Ratios can be obtained by exponentiating the ordered logit coefficients, ${e}^{coef}.$ For a one unit change in the predictor variable, the odds for cases in a group that is greater than k versus less than or equal to k are the proportional odds times larger, where k is the level of the response variable. Therefore, as the coefficients of all red and red timings were negative, with one unit increase in all red and red (when other variables are constant), the odds of low PET meaning high risk values are 1.188 and 0.986 times larger respectively. So, for yellow, red clearance and green timings, as these coefficients are positive, with one unit increase in yellow, red clearance and green timings (when other variables are constant in each of the models), the odds of values in high PET meaning low risk levels are 1.109, 1.034 and 1.015 times larger respectively. This leads to an important conclusion regarding improving the PETs at intersections. Increasing the yellow, red clearance and green timings would lead to better PETs than increasing all red and red time. Since the data collected was during the afternoon peak, it might also be impactful to increase yellow and red clearance time for these periods only rather than for the entire length of day.

### Heterogeneity analysis

From Table 3, yellow time was found to be significant normally distributed random parameters with mean of 0.014 and standard deviation of 0.363. Therefore, larger yellow times are associated with higher PET levels meaning less critical conflicts. Intersection variable is also normally distributed with a mean of 7.445 and standard deviation of 4.824. This means that vehicles within an intersection are more likely to be associated with higher levels of PET during all red time. An intersection is a less critical conflict location than an approach during all red time. The same can be said about red clearance time since the intersection variable was also statistically significant with a mean of 1.8 and standard deviation of 1.6. Red time was also significant with a mean of − 0.014 and standard deviation of 0.007. Therefore, more critical conflicts are noticed at the start of red time. This is expected since at the start of red time, the vehicles slow down and come to a complete stop. Therefore, no unsafe PET levels are observed at the end of red time. The volume variable was also significant during red time (mean − 0.023, standard deviation 0.014). Higher volumes during red time are related to lower PET levels giving rise to more critical conflicts. Similarly, higher volume during green time also results in more critical conflicts (mean − 0.003 and standard deviation 0.006). The volume was not significant during the yellow, red clearance and all red time but was significant during the longer durations such as green and red time.

## Conclusions

In summary, this paper proposes the use of UAV vehicle trajectory data to identify the relationship between signal timing and PET. One hour of UAV data was collected to obtain PETs, speeding, heading and signal phasing and timing. The PETs were calculated using rotating bounding boxes and also using the back of the leading vehicle and front of the lagging vehicle which gives a much accurate PET than that using center points of the vehicles. It was then modelled using Random Parameter Ordered Logit Model with heterogeneity in means. The PET values were divided into five classes. Results from the model showed that the yellow time and red clearance time are negatively related with PET while all red time, red time and green time are positively related to PET. The odds ratio indicated that it would be possible to increase the PET levels and thereby improve safety by only increasing the yellow time and red clearance time by 1 s. The practical application of this study can be achieved in signal timing optimization. Usually, the various times are decided based on traffic volume and intersection geometry only. Safety remains largely disregarded. Using the results from this study, the signal timing can be optimized based on safety parameters also so that less conflicts are expected.

This study can be used to understand the safety of an intersection in terms of signal timing. Following distance was calculated to indicate aggressive driving behavior and how it varies with the different phases. The results showed that drivers tend to follow closely during the end of yellow and during all red time. It can also assist in determining if signal retiming is warranted to help improve safety. Only an hour of video data processing has the potential to provide these insights to relevant authorities. Future studies can focus on the traffic dynamic features as well as different types of intersections to understand the relationship between surrogate safety measures and signal timing. Moreover, other measures of conflicts such as time to collision (TTC), modified time to collision (MTTC), deceleration rate to avoid a crash (DRAC), etc. can also be studied.

## Data Availability

The datasets used during the current study are available on GitHub: https://github.com/ozheng1993/UCF-SST-CitySim-Dataset.
